# Reproductive health and pregnancy outcomes among French gulf war veterans

**DOI:** 10.1186/1471-2458-8-141

**Published:** 2008-04-28

**Authors:** Catherine Verret, Mathe-Aline Jutand, Catherine De Vigan, Marion Bégassat, Lynda Bensefa-Colas, Patrick Brochard, Roger Salamon

**Affiliations:** 1 Laboratory of Occupational and Environmental Health, Victor Segalen Bordeaux 2 University, Bordeaux, France; 2 Département d'Epidémiologie et de Santé Publique Nord, Ecole du Val-de Grâce, Paris, France; 3 Epidemiology, Public Health and Development (INSERM U593), Victor Segalen University Bordeaux 2, Bordeaux, France; 4 Paris Registry of Congenital Malformations, INSERM UMR S149, IFR 69 Villejuif, France, Université Pierre et Marie Curie 6, Paris, France; 5 Clinic of Occupational Diseases, Cochin Hospital, AP-HP, Paris 5 University, Paris, France

## Abstract

**Background:**

Since 1993, many studies on the health of Persian Gulf War veterans (PGWVs) have been undertaken. Some authors have concluded that an association exists between Gulf War service and reported infertility or miscarriage, but that effects on PGWV's children were limited.

The present study's objective was to describe the reproductive outcome and health of offspring of French Gulf War veterans.

**Methods:**

The French Study on the Persian Gulf War (PGW) and its Health Consequences is an exhaustive cross-sectional study on all French PGWVs conducted from 2002 to 2004. Data were collected by postal self-administered questionnaire. A case-control study nested in this cohort was conducted to evaluate the link between PGW-related exposures and fathering a child with a birth defect.

**Results:**

In the present study, 9% of the 5,666 Gulf veterans who participated reported fertility disorders, and 12% of male veterans reported at least one miscarriage among their partners after the PGW. Overall, 4.2% of fathers reported at least one child with a birth defect conceived after the mission. No PGW-related exposure was associated with any birth defect in children fathered after the PGW mission. Concerning the reported health of children born after the PGW, 1.0% of children presented a pre-term delivery and 2.7% a birth defect. The main birth defects reported were musculoskeletal malformations (0.5%) and urinary system malformations (0.3%). Birth defect incidence in PGWV children conceived after the mission was similar to birth defect incidence described by the Paris Registry of Congenital Malformations, except for Down syndrome (PGWV children incidence was lower than Registry incidence).

**Conclusion:**

This study did not highlight a high frequency of fertility disorders or miscarriage among French PGW veterans. We found no evidence for a link between paternal exposure during the Gulf War and increased risk of birth defects among French PGWV children.

## Background

In late 1990 and early 1991, approximately 20,000 French troops were deployed in the First Gulf War. Since 1993, many studies on the health of Persian Gulf War veterans (PGWVs) have been undertaken, mainly in the USA and the UK. US and UK mortality studies have shown a lower mortality from all illnesses among Gulf War veterans in comparison to non-deployed veterans and an increased mortality from external causes consistent with patterns of postwar mortality observed in veterans of previous wars [1-4]. Compared to non-deployed veterans, PGWVs have been reported to have no excess of recognized diseases [5-17] but were 2–3 times more likely to report fatigue, cognitive difficulties, headaches, myalgia and arthralgia, mood disturbances, and sleep problems [5-17].

Several studies have been conducted on reproductive outcomes [18-31], mainly focused on pregnancy outcomes and birth defects [18-25,27,29-31], but no specific studies on spermatogenesis anomalies.

One study did not find any difference between the reproductive hormones measured in Gulf War veterans and in controls [24]. However, compared with non-deployed controls, Gulf War veterans had a significantly higher risk of reported infertility (OR: 1.4 to 1.5 according to the fertility type) [28] and a higher risk of self-reported sexual problems (OR: 3.5 – p < 0,001) [24].

Pregnancies fathered by Gulf War veterans took longer to conceive [28]. Conceptions of exposed and nondeployed veterans have similar outcomes [20,23,26], and no differences in pre-term deliveries are described [25,29,30]. Female Gulf War veterans had a higher risk of ectopic pregnancies than non-deployed veterans [20]. The increase of miscarriage or spontaneous abortion was not consistent between the different studies [20,22-26]. Compared with non-deployed veterans, Gulf War veterans reported the same risk of stillbirth [23,25,26] or infant mortality [25].

Most of the studies on the children of Gulf War veterans found no evidence of an increase in the risk of birth defects [18,21,22,24,29,31] or congenital diseases [22,24]. Some authors reported a higher prevalence of reported birth defects in babies of Gulf War veterans conceived after the Gulf War [22,23,25]. When looking at specific defects, only Araneta *et al *[19] observed a higher prevalence of renal agenesis and hypoplasia among children conceived postwar to GWV men, adjusted for prenatal alcohol exposure and intrauterine growth retardation. The other birth defects described (cardiac valve disorder among children conceived postwar to GWV men and hypospadias among children conceived postwar to GWV women) were no longer significant after adjustment for maternal parameters, branch of military service, and military rank [19].

For Doyle et al [32], there is no strong or consistent evidence in the literature of an effect of paternal service in the first Gulf War on the risk of major birth defects or stillbirth in offspring conceived after deployment, even if effects on specific rare defects cannot be excluded. There is some evidence of small increased risks of miscarriage or infertility associated with service, but the role of bias cannot be ruled out [32]. Finally, with regards to female veterans, firm conclusions cannot be drawn due to lack of sufficient information [32].

The present study reports findings relating to the reproductive outcome and health of offspring of French Gulf War veterans. The French Study on the Persian Gulf War and its Health Consequences is an exhaustive investigation into all French PGWVs conducted from 2002 to 2004. The aim of this descriptive study was, mainly, to examine self-reported symptom data among Gulf War veterans and to describe the main exposures reported in the theater, the symptoms and diseases that appeared during and after the Persian Gulf mission, and children's health.

## Methods

### Population study

Detailed information about this study is given elsewhere [33]. In brief, the French Study on the Persian Gulf War and its Health Consequences is a cross-sectional study with an exhaustive aim which included all civilians and military personnel serving in the Persian Gulf from August 1990 to July 1991. The addresses of 10,478 French troops who served in the Gulf during the period from August 1990 through July 1991 were identified, based on data transmitted by military staff, and 5,666 participated in the study after receiving two reminders. The participation rate was 54% and varied by branch of service (56% in the Army, 55% in the Air Force, and 43% in the Navy).

### Questionnaire

Data collection was based on a 12-page self-administered postal questionnaire, accompanied by an explanatory letter stating the objectives of our study and a consent form, was developed on the basis of information published in the French authorities' reports and with reference to questionnaires used in PGWV morbidity studies. The questionnaire, tested on a sample of the target population, requested details of disorders requiring medical consultation, miscarriage or stillbirth, and the number of children born before and after the conflict. If a child presented a disease, his or her year of birth, gender, and detailed illnesses were requested. The notion of infertility was inferred from the following specific disorders: infertility or sperm abnormalities. The questionnaire also explored (i) socio-demographic characteristics (gender, age), (ii) military history (service branch, rank, military status on completion of the questionnaire), (iii) living conditions, and self-reported exposures during the Persian Gulf mission (sandstorms, smoke from oil well fires, chemical or bacteriological alerts, vaccinations, medication, and pesticides) of the PGWVs, and (iv) diseases and symptoms before, during, and after the mission. Hospitalization after the mission for one of the 49 symptoms of the Hopkins Symptom Checklist [34] was also reported.

On reception, the questionnaires were made anonymous, coded by an epidemiologist (CV) [ICD-10 [35]], keyboarded, and analyzed. Birth defects presented by PGWV offspring were grouped for analysis based on the classification system used in the European Registry of Congenital Anomalies (EUROCAT) [36].

### Statistical analysis

We used Stata™ statistical software for all analyses. All p values are two sided, and we took values less than 0.05 to indicate statistical significance.

First, a descriptive analysis was conducted on PGWV: i) living conditions and exposures during the mission; ii) self-reported infertility disorders and miscarriage that appeared after the PGW mission.

Secondly, as data on healthy children were not available (except for the number of children), we decided to perform a case-control study nested within the cohort to determine the effect of PGW exposures on the risk of a father conceiving a child with a birth defect after the war. The date of birth had to be later than nine months after the end of the PGW mission for a child to be considered as having been conceived after the war. A case was defined as a man having had at least one child conceived after the PGW mission and presenting at least one birth defect. Two controls were selected for each case. A control was defined as a man of the same age as the case (± 1 year) who had never had a child with a birth defect but who had had at least one child after the PGW mission. Fathers having had at least one child with a birth defect before the PGW were excluded from this analysis. To minimize recall bias, a control should have reported one hospitalization for at least one symptom of the Hopkins Symptom Checklist [34] after the mission. PGW exposure odds-ratios were estimated by conditional logistic regression and then adjusted for service branch, rank, and military status.

Finally, the study provides a description of children born to PGWVs after the Gulf War. Major anomalies were described as Maconochie et al reported [27]. Minor anomalies were coded and specified. As no national registry on birth defects has been developed in France, PGWV children's birth defect rates were compared to the 10-year incidence rate of birth defects among children conceived from 1991 to 2000, as described by the Paris Registry of Congenital Malformations [37]. The Paris Registry rate was considered as the reference rate among the population. The confidence interval of the PGWV children's birth defect rate was estimated according to a Poisson distribution. The incidence ratio was calculated by dividing the PGWV children's birth defect rate by the reference rate.

The National Commission of Data Processing and Civil Liberty approved this investigation, in conformity with article 15, paragraph 3 of the Law of January 6, 1978, concerning data processing, files, and civil liberty.

## Results

### Study population

Most of the 5,666 subjects who completed the questionnaire were male (99.5%), with an average age of 41 years (SD: 6 years) at the time of completion of the survey, and 71% of respondents were still in service. Considering the sample size of female veterans, the results are presented below by gender.

### Reproductive health of French female Persian Gulf War veterans

The mean age of the 28 women who completed the questionnaire was 44 years (37 to 57 years). They served mainly in military health services (n = 14) and in the Air Force (n = 12). One woman served in the Navy and one in the Army. Six women reported at least one miscarriage after the mission. Nine women reported having at least one child after the mission (total of 25 babies), ranging from 1 to 4 children. No birth defect was reported in children born after the mission.

### Reproductive health of French male Persian Gulf War veterans

The main characteristics of the 5,638 male veterans are presented in Table 1. Respondents served mainly in the Army or the Air Force and were servicemen. The respondents' age varied according to service: 63% of Army veterans, 52% of Navy veterans, and 28% of Air Force veterans were less than 40 years old (p < 0,001).

**Table 1:** Main Characteristics and Reproductive History of French Male Persian Gulf War Veterans (N = 5,638)

	Army (n = 2,694)		Navy (n = 769)		Air Force (n = 1,883)		Other branch* (n = 292)		Total (n = 5,638)	
	n	%	n	%	n	%	n	%	n	%
**Main characteristics**										
Rank										
Officers	277	10.3	101	13.1	364	19.3	120	41.1	862	15.3
Non-commissioned officers	962	35.7	435	56.6	1,431	76.0	122	41.8	2,950	52.3
Servicemen	1,455	54.0	233	30.3	88	4.7	50	17.1	1,826	32.4
Age (at the beginning of the study)										
< 40 years	1,699	63.1	401	52.1	528	28.0	68	23.3	2,696	47.8
>= 40 years	995	36.9	368	47.9	1,355	72.0	224	76.7	2,942	52.2
Military status										
Active	1,980	73.5	668	86.9	1,202	63.8	190	65.1	4,040	71.7
Retired	714	26.5	101	13.1	681	36.2	102	34.9	1,598	28.3
										
**Reproductive history after the PGW mission**										
Fathers having at least one child (live birth)	1,706	63.3	436	56.7	853	45.3	126	43.2	3,121	55.4
*at least one conceived before and after GW*	*406*	*15.1*	*132*	*17.2*	*354*	*18.8*	*63*	*21.6*	*955*	*16.9*
										
Number of live births per father (after the mission)										
0	988	36.7	333	43.3	1,030	54.7	166	56.8	2,517	44.6
1	719	26.7	190	24.7	382	20.3	61	20.9	1,352	24.0
2	710	26.4	175	22.8	344	18.3	40	13.7	1,269	22.5
>= 3	227	8.4	55	7.2	91	4.8	20	6.8	393	7.0
										
Any miscarriage or stillbirth	395	14.7	96	12.5	170	9.0	21	7.2	682	12.1
*at least one before and after GW*	*16*	*0.6*	*5*	*0.7*	*9*	*0.5*	*3*	*1.0*	*33*	*0.6*
										
Infertility reported	35	1.3	5	0.7	6	0.3	2	0.7	48	0.9
										
Father with child born with birth defects	69	2.6	15	2.0	45	2.4	6	2.1	135	2.4
*at least one before and after GW*	*0*	*0.0*	*2*	*0.3*	*2*	*0.1*	*0*	*0.0*	*4*	*0.1*

* Other branch of service: state public force, medical military service

#### Infertility and other reproductive outcomes

Infertility and reproductive outcomes are described in 5,638 male veterans. Infertility problems (such as spermogram anomalies) were reported by 48 respondents, mainly Army veterans (Table 1).

After deployment, 3,121 veterans fathered at least one child (corresponding to 5,158 babies). The number of pregnancies reported per father varied from 1 to 10 pregnancies.

After deployment, 682 male veterans (12%) reported at least one miscarriage by their partner, 33 of whom reported a miscarriage before and after the mission. Overall, 135 men (4.3% of fathers) reported at least one child born with at least one birth defect (140 children), of which 4 reported having one child presenting a birth defect both before and after the mission (8 children). After the PGW mission, 131 fathers (4.2%) reported at least one child born with a birth defect, without having conceived a child with a birth defect before the PGW mission.

#### Case-control study of PGW-related exposures on any birth defects

The case-control study of PGW-related exposures on any birth defects included 131 cases and 262 controls. The 262 controls were randomized among the 845 veterans who had reported one hospitalization for at least one symptom (14.9% of participants), and matched for age (+/- one year). Characteristics of subjects in terms of branch of service, rank and military status, and crude and adjusted odds ratios for any birth defects are presented in Table 2.

**Table 2:** Crude and Adjusted Odds Ratios for Any Birth Defect – Role of Service, Rank, Military Status

	Cases (n = 131)		Controls (n = 262)		Univariate analysis			Adjusted analysis *		
						
	n	%	n	%	OR	95% CI	P	OR	95% CI	p
Age in 1990					1.02	0.83–1.25	0.84	1.02	0.83–1.27	0.81
										
Service							0.06			0.08
Navy	13	9.9	24	9.2	1.00			1.00		
Army	69	52.7	169	64.5	0.75	0.35–1.62		0.74	0.33–1.61	
Air Force	43	32.8	58	22.1	1.69	0.70–4.07		1.72	0.71–4.17	
Other branch	6	4.6	11	4.2	1.23	0.31–4.91		1.16	0.28–4.77	
										
Rank							0.60			0.60
Officers	15	11.5	24	9.2	1.00			1.00		
Non-commissioned officers	58	44.3	114	43.5	0.77	0.36–1.65		0.69	0.31–1.56	
Servicemen	58	44.3	124	47.3	0.64	0.27–1.54		0.86	0.33–2.21	
										
Military status										
Active	99	75.6	191	72.9	1.00			1.00		
Retired	32	24.4	71	27.1	0.87	0.53–1.41	0.57	0.82	0.49–1.36	0.44

* adjusted odds ratio for service branch, rank, and military status

Cases did not differ from controls according to service branch, rank, or military status.

Description of PGW-related exposures and odds ratios for any birth defects adjusted for branch of service, rank, and military status are presented in Table 3.

**Table 3:** Crude and Adjusted Odds Ratios for Any Birth Defect – Role of PGW-Related Exposures

	Cases (n = 131)		Controls (n = 262)		Univariate analysis			Adjusted analysis *		
						
	n	%	n	%	OR	95% CI	p	OR	95% CI	p
Mission in GW							0.17			0.31
Before Jan. 1991	12	9.2	35	13.4	1.00			1.00		
After Feb. 1991	6	4.6	20	7.6	0.85	0.28–2.58		0.85	0.27–2.66	
Only Jan-Feb. 1991	17	13.0	19	7.3	2.33	0.97–5.59		1.88	0.75–4.75	
Other date of mission	96	73.3	188	71.7	1.43	0.70–2.91		1.61	0.76–3.40	
										
Location of mission							0.47			0.90
Neither Iraq nor Kuwait	72	55.0	122	46.6	1.00			1.00		
Kuwait only	10	7.6	23	8.8	0.73	0.32–1.66		0.74	0.30–1.78	
Iraq only	41	31.3	96	36.6	0.72	0.44–1.17		0.97	0.53–1.78	
Iraq and Kuwait	8	6.1	21	8.0	0.66	0.28–1.58		0.84	0.33–2.10	
										
Smoke of oil well fires										
No	90	68.7	176	67.2	1.00			1.00		
Yes	41	31.3	86	32.8	0.93	0.59–1.47	0.76	0.90	0.56–1.43	0.65
										
Sandstorm										
No	25	19.1	49	18.7	1.00			1.00		
Yes	106	80.9	213	81.3	0.98	0.58–1.65	0.93	1.15	0.57–2.31	0.69
										
Chemical alarms										
No	46	32.1	66	25.2	1.00			1.00		
Yes	85	64.9	196	74.8	0.63	0.40–0.99	0.05	0.61	0.36–1.06	0.08
										
Pesticides										
No	86	65.7	178	67.9	1.00			1.00		
Yes	45	34.4	84	32.1	1.11	0.70–1.78	0.64	1.00	0.61–1.63	0.99

* adjusted odds ratio for age, service branch, rank, and military status

Service period and locations for the PGW mission (Iraq and/or Kuwait) were not different for cases and controls. Concerning PGW-related exposures, cases reported an identical exposure to the smoke of oil well fires, sandstorms, chemical alarms, and pesticides. Controls more often reported an exposure to sounding of chemical alarms (74.8% *vs *64.9%), which did not persist after adjustment for service branch, rank, and military status.

### Health of live born children

The health of live born children and birth defects are described in 5,183 children conceived after the PGW mission. Incidence of pre-term delivery reported in children conceived after the PGW mission was 104.2/10,000 live births [95% CI: 78.3–135.9]. Only 140 babies conceived after the PGW mission presented at least one birth defect (270.1/10,000 live births [95% CI: 227.2–318.7]). Eight babies presented two malformations. These associations were not specific.

The different major birth defects of babies conceived after the Gulf War mission are presented in Table 4.

**Table 4:** Major Birth Defects* among French PGWV's Children Born after the Mission (N = 5,183)

	Children born in		All children		
		
	1991–1992	1993–2001	Total	Rate (p.10,000)	[95%CI]
Any major birth defect	21	119	140	270.1	[227.2–318.7]
					
Central nervous system	2	4	6	11.6	[4.2–25.2]
Neural tube defects	1	2	3	5.8	[1.2–16.9]
Hydrocephalus	0	1	1	1.9	[0.1–10.8]
Other malformation of central nervous system	1	1	2	3.9	[0.5–13.9]
					
Eye, ear, face and neck	1	8	9	17.4	[7.9–33.0]
Eye	1	3	4	7.7	[2.1–19.8]
Ear	0	2	2	3.9	[0.5–13.9]
Other malformation of eye, ear, face, nose	0	3	3	5.8	[1.2–16.9]
					
Circulatory system	2	13	15	28.9	[16.2–47.7]
Congenital malformation of heart	0	12	12	23.2	[12.0–40.4]
Other malformation of circulatory system	2	1	3	5.8	[1.2–16.9]
					
Respiratory system	1	2	3	5.8	[1.2–16.9]
					
Cleft lip/palate	0	3	3	5.8	[1.2–16.9]
					
Digestive system	3	10	13	25.1	[13.4–42.9]
TOF and other malformations of large intest., rectum, anal canal	0	3	3	5.8	[1.2–16.9]
Other malformation of digestive system	3	7	10	19.3	[9.3–35.5]
					
Genital system	2	8	10	19.3	[9.3–35.5]
					
Urinary system	2	16	18	34.7	[20.6–54.9]
Renal anomalies	0	7	7	13.5	[5.4–27.8]
Urinary tract anomalies	2	9	11	21.2	[10.6–38.0]
					
Musculoskeletal system	4	20	24	46.3	[29.7–68.9]
Limb reduction	0	2	2	3.9	[0.5–13.9]
Polydactyly and syndactyly	0	3	3	5.8	[1.2–16.9]
Other limb malformation	1	11	12	23.2	[12.0–40.4]
Anomalies of diaphragm, exomphalus, gastrochisis	0	3	3	5.8	[1.2–16.9]
Other musculoskeletal anomalies	3	3	6	11.6	[4.2–25.2]
					
Other specific non-chromosomal syndromes	2	7	9	17.4	[7.9–33.0]
Specified syndromes (non-chromosomal)	0	3	3	5.8	[1.2–16.9]
Other non-chromosomal malformations	2	4	6	11.6	[4.2–25.2]
					
Chromosomal	2	12	14	27	[14.8–45.3]
Down syndrome	2	4	6	11.6	[4.2–25.2]
Other chromosomal	0	8	8	15.4	[6.7–30.4]

* excluded: minor or unspecified anomalies of ear, undescended testes, unspecified congenital deformity of hip, unspecified congenital deformity of feet, naevus

The main birth defects reported in babies conceived after the mission were anomalies of the musculoskeletal system (rate: 46.3/10,000) and urinary system anomalies (34.7/10,000). However, no specific birth defect was found. Only 21 children conceived in the first two years following the conflict presented birth defects, mainly malformations of the digestive system.

### Comparison with the Paris Registry of Congenital Malformations

**Figure 1:**
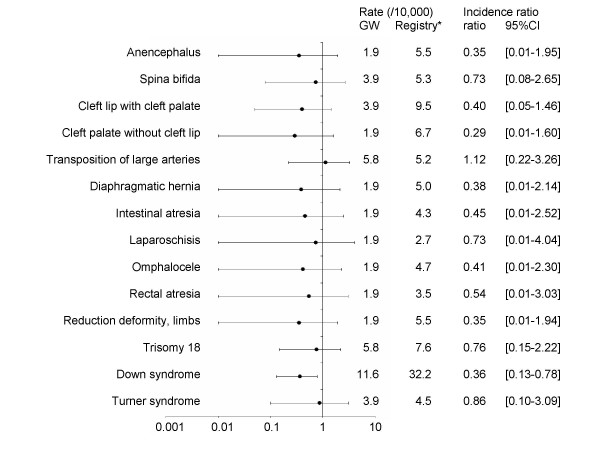
**Rates of Congenital Malformations in PGWV' children and in the Paris Registry of Congenital Malformations**. * 10-year incidence

The birth defect frequencies estimated after deployment were compared to available French data (Paris Registry of Congenital Malformations, 1991–2000). The 10-year birth defect incidence rate of children conceived after deployment (270.1/10,000 [227.2–318.7]) was not different from the incidence of the birth defect Registry (261.0/10,000 [255.8–266.2]; ratio: 1.03 [95%CI: 0.9–1.2]). Figure 1 presents the incidence ratios of the main birth defects and their 95% confidence intervals.

Compared with the Registry birth defect incidence, no main birth defect was higher in the veterans' children group. Only Down syndrome incidence was statistically lower in the veterans' children group than in the Registry one (rate of veterans' children group: 11.6/10,000 [95% CI: 4.2–25.2] – Registry: 32.2/10,000 [30.4–34.1]; incidence ratio: 0.4 [95%CI: 0.1–0.8]).

## Discussion

In this study, 1% of the Gulf veterans reported fertility disorders and 12% of male veterans reported at least one miscarriage among their partners after the PGW mission. Overall, 4.2% of fathers reported at least one child born with a birth defect after the mission. No PGW-related exposure was associated with any birth defect in children fathered after the PGW mission. Concerning the reported health of children born after the PGW mission, 1.0% of children were born with pre-term delivery and 2.7% presented a birth defect. The main birth defects reported were musculoskeletal malformations (0.5%) and urinary system malformations (0.3%). Birth defect incidence in PGWV children conceived after the mission was similar to birth defect incidence described by the Paris Registry; except for Down syndrome, where the incidence in PGWV children was lower than the Paris Registry incidence.

Results are reported with several reservations. Although we sent out two reminders, our study did not have a high response rate, which suggests selective participation by respondents according to health outcome. The French Study on the Persian Gulf War and its Health Consequences aimed to be exhaustive, offering a free medical examination to all veterans provided by the French Government. The media in foreign countries gave massive coverage to complaints lodged due to the health consequences of the Gulf War, so respondents with health disorders, or at least worried about them, were more willing to participate in our study. However, this bias could be partly compensated for by the fact that 71% of respondents were still in service.

Data were gathered retrospectively and were based on veterans' self-reported data and were not validated against medical records for healthy veterans or their children. However, it is probable that symptoms or serious diseases requiring specific care or of unusual frequency or intensity were reported more often than events considered as slight or benign.

Recall bias can be a serious problem in case-control studies; when pre-recorded written exposure information is unavailable, controls with identical stimuli should be selected (e. g. a control series of children with malformations other than the one under study) [38]. We conducted a case-control study including controls matched for age and who reported one hospitalization for at least one symptom in order to minimize the recall bias.

A direct relationship of birth defects to characteristics of the PGW mission could not be shown because i) data were collected retrospectively on a 10-year period, ii) information was based on the declarations of the PGW veterans (most were men, with no information on the children's mother), and iii) this study can only highlight associations between exposures and diseases and not evidence. Moreover, many children were conceived long after the war itself, personal risk factors (i.e., family history or history of another child with a birth defect) were not examined, and there is a potential for misreporting. No information was available on the use of medication. Results must be interpreted with caution due to the small number of case children for specific birth defects. Despite these major limitations, frequencies shown in French PGWVs were similar to frequencies in the French general population, except for the low frequency of Down syndrome. In our study, dates of birth of the unaffected children were not available and fathers' ages at birth were not known. However, 82% of fathers were 25 to 35 years old during the period covered by the study. Paternal age is related to maternal age and the risk of infecundity, miscarriage, and birth defects seems to increase with paternal age [39,40]. In the registry, 22% of mothers were above 35 years of age [37]. The analysis could not be controlled for mothers' age or parity.

The results of fertility studies on Gulf War veterans are controversial. The first studies published [24,28] did not find any effect of Gulf War service on markers of male fertility (hormone measurements, oligospermia, azoospermia, asthenospermia, teratospermia, sexual problems). However, some authors described an increased risk of infertility reaching 7% to 14% [25,26], mainly due to an increase of teratospermia and oligoasthenospermia [28]. The low estimation (0.9%) in our study could be explained by the self-reported infertility data collected in this study. Besides these sperm anomalies, pregnancies fathered by PGWVs seemed to take longer to conceive [27].

Authors reported an increased risk of miscarriage among partners of PGWVs, compared to a control group [22,23,25-27], ranging from 12% to 60% among first pregnancies conceived after the PGW mission [25]. In our study, this miscarriage rate was 12% after the mission.

Concerning children's health in our study, the frequency of pre-term delivery (1.0%) was lower than frequencies described in other studies on PGW veterans (ranging from 0.6% to 10.9%) [18,19,23,25,26,30].

In our study, the frequency of birth defects was slightly lower (2.7%) than the frequencies described in other studies (ranging from 3.6 to 9.0%) [21-23,25,26]. The rate of birth defects among the French general population is estimated at 3.2% of all births for the period 1981–2000 [37]. No specific birth defect was highlighted in our study. Moreover, as one hypothesis was that wartime exposures adversely affected spermatogenesis, it was therefore reasonable to evaluate conceptions that occurred 70–90 days after leaving the war environment. The assessment of conceptions that occurred 3–10 years after the PGW mission would be more a reflection of non-war exposures and advanced paternal age than of distant wartime exposures. Since the number of live births per year was not available, in order to show a statistical difference between the birth defect rates of each of the two periods (1991–1992 *vs *1993–2001), we estimated by simulation that fewer than 700 live births were needed in 1991–1992 (i.e. 13% of all children born after the PGW mission). However, this figure did not seem plausible in view of the context (a return home after an overseas mission). Specific birth defect frequencies were lower in our study than frequencies described by Doyle [22] in UK PGWVs: malformations of the musculoskeletal system (8.0‰ in Doyle's study *vs *4.6‰ in our study), or malformations of the urinary system (4.9‰ in Doyle's study *vs *3.5‰ in our study). The frequency of renal anomalies (1.4‰) was similar to that described by Araneta [19] in US PGWVs (1.1‰), and chromosomal anomalies were similar to those previously described (2.7‰ in our study, 0.2‰ to 2.6‰ in the literature) [19,25]. Since 1970, the increase in malformations of the urinary, central nervous, and cardiac systems could be explained by the widespread use of ultrasound, (particularly in antenatal diagnosis), which can identify malformations even without clinical symptomatology.

A combination of genetic and environmental factors may be responsible for 20 to 25% of congenital anomalies [41]. US Gulf veterans were exposed to many chemical, biological, and physical agents suspected of being reproductive toxins [42]. Constant infertility over time of UK veterans, described by Maconochie [28], argues in favor of either paternal germ cell mutation or other damage to spermatogenic stem cells necessary for supporting spermatogenesis. Combined exposure to pyridostigmine bromide, the insect repellent DEET, and the insecticide permethrin seemed to induce apoptosis in rat testicular germ cells, Sertoli cells, and Leydig cells [43]. However, Arfsten [44] showed that implantation of depleted uranium in adult rats does not have an adverse impact on male reproductive success, sperm concentration, or sperm velocity. Our case-control analysis did not highlight the role of PGW-related exposures in birth defects among PGWV children.

## Conclusion

In conclusion, this study found the same frequencies of fertility disorders, miscarriage, and health disorders among PGWV children as those described among foreign PGWVs and the French general population. No PGW-related exposure was associated with any birth defect in children fathered after the PGW mission. Our findings are limited by the reliability of self-reported data concerning exposures and health and pregnancy outcomes. This study highlights the importance of prospective data collection for exposures during future foreign operations and epidemiological surveillance of servicemen and women.

However, if the fertility disorders and birth defects remain constant over time, a more detailed and focused survey would be required to examine fertility and other aspects of reproduction more thoroughly.

## Competing interests

The French Study on Persian Gulf War and its Health Consequences was funded by the French Ministry of Defense.

## Authors' contributions

CV carried out study design, statistical analyses and drafting the manuscript, MAJ supervised all aspects of study design and statistical analyses, CDV helped to findings interpretation on birth defects analyses, MB participated in study design and carried out data collection, LBC helped revise the manuscript, PB supervised analyses and helped revise the manuscript, RS was the principal investigator and supervised all aspects of study implementation. All authors read and approved the final manuscript.

## Pre-publication history

The pre-publication history for this paper can be accessed here:


